# Electroacupuncture Reduces the Effects of Acute Noxious Stimulation on the Electrical Activity of Pain-Related Neurons in the Hippocampus of Control and Neuropathic Pain Rats

**DOI:** 10.1155/2016/6521026

**Published:** 2016-10-19

**Authors:** Jun-Ying Wang, Renbo Chen, Shu-Ping Chen, Yong-Hui Gao, Jian-Liang Zhang, Xiu-Mei Feng, Yaxia Yan, Jun-Ling Liu, Ingrid Gaischek, Daniela Litscher, Lu Wang, Irmgard Th. Lippe, Gerhard Litscher

**Affiliations:** ^1^Department of Physiology, Institute of Acupuncture and Moxibustion, China Academy of Chinese Medical Sciences, Beijing 100700, China; ^2^Research Unit for Complementary and Integrative Laser Medicine, Research Unit of Biomedical Engineering in Anesthesia and Intensive Care Medicine, and TCM Research Center Graz, Medical University of Graz, 8036 Graz, Austria; ^3^Department of Chronic Diseases, Institute of Basic Research in Clinical Medicine, China Academy of Chinese Medical Sciences, Beijing 100700, China; ^4^Institute of Experimental and Clinical Pharmacology, Medical University of Graz, 8036 Graz, Austria

## Abstract

To study the effects of acupuncture analgesia on the hippocampus, we observed the effects of electroacupuncture (EA) and mitogen-activated protein kinase (MEK) inhibitor on pain-excited neurons (PENs) and pain-inhibited neurons (PINs) in the hippocampal area CA1 of sham or chronic constrictive injury (CCI) rats. The animals were randomly divided into a control, a CCI, and a U0126 (MEK1/2 inhibitor) group. In all experiments, we briefly (10-second duration) stimulated the sciatic nerve electrically and recorded the firing rates of PENs and PINs. The results showed that in both sham and CCI rats brief sciatic nerve stimulation significantly increased the electrical activity of PENs and markedly decreased the electrical activity of PINs. These effects were significantly greater in CCI rats compared to sham rats. EA treatment reduced the effects of the noxious stimulus on PENs and PINs in both sham and CCI rats. The effects of EA treatment could be inhibited by U0126 in sham-operated rats. The results suggest that EA reduces effects of acute sciatic nerve stimulation on PENs and PINs in the CA1 region of the hippocampus of both sham and CCI rats and that the ERK (extracellular regulated kinase) signaling pathway is involved in the modulation of EA analgesia.

## 1. Introduction

Peripheral nerve injuries produce acute pain and often induce neuropathic pain, a severe clinical problem and chronic debilitating condition that affects the nervous system. Neuropathic pain is relatively common and impairs the quality of life of sufferers. In the past few decades, neuropathic pain animal models have been used to study pain mechanisms and analgesia effects. Chronic constrictive injury (CCI) has been the common neuropathic pain model since 1988 [1, 2].

The hippocampus, a part of the limbic system, has the function of learning, memory, emotion, and affect and also has relationships with chronic and acute pain. It has been reported that hippocampal formation plays an important role in pain information processing, including anatomical features, behavioral experiments, electrophysiology, functional imaging, and other molecular research [3]. Humans suffering from chronic or severe pain had smaller hippocampal volumes. Hippocampal N-acetylaspartate/creatine decreased in elderly patients suffering from acute pain [4, 5]. Neuropathic pain induced hippocampal interleukin-1 beta (IL-1*β*) mRNA levels upregulation, and the changes of IL-1*β* mRNA expression correlated with the injured side of the hippocampus [6]. Xiao et al. [7], Yang et al. [8], and Li et al. [9] reported in a series of studies that acetylcholine (ACh) influences the pain-induced discharge frequency and the electric activities of pain-related neurons in the hippocampus of rats.

Acupuncture has been widely applied in China and other Asian countries for thousands of years to ameliorate a number of diseases, including acute and chronic pain. Acupuncture analgesia has been gradually accepted by people due to its advantages, but its mechanism has not yet been clarified in detail. Mounting evidence from many laboratories over the past years suggests that acupuncture has effects on the hippocampus. Traditional acupuncture treatments significantly decreased functional magnetic resonance imaging (fMRI) signals [10] and the metabolism in the hippocampus [11]; 2 Hz electrical acupoint stimulation-induced analgesia has negative correlations with the averaged fMRI activation levels of the bilateral hippocampus [12]. Electroacupuncture (EA) could modulate the function of interneurons in the hippocampus, significantly enhancing long-term potentiation (LTP) [13]. In some of our previous studies, chronic pain had an effect on the hippocampal cholinergic system, and EA treatment relieved pain by regulating hippocampal cholinergic neurons [14]. EA had an obvious analgesic effect and in CCI rats significantly diminished the injury-induced increase in synaptic cleft width and thinning of the postsynaptic density [15, 16]. It also activated the extracellular regulated protein kinases (ERK) signaling pathway in the hippocampus [17]. Previous studies suggested that ERK/mitogen-activated protein kinase (MEK) play an essential role in neuropathic pain [18]. In the spinal cord, CCI-induced neuropathic pain activated the ERK- and cAMP-response element binding protein (CREB) signaling pathway [19]. However, it remains largely unknown how the ERK is involved in pain modulation in the hippocampus.

On the basis of these studies, the objective of this research is to investigate the effects of EA and the ERK signaling pathway on the acute pain-induced responses of pain-related neurons in the hippocampus of Wistar rats under both control and neuropathic pain conditions.

## 2. Materials and Methods

### 2.1. Animals and Groups

Adult male Wistar rats, weighing from 220 g to 280 g (*n* = 43), purchased from Beijing Union Medical College, were acclimatized to standard laboratory conditions (12-hour light and dark cycle) at the Beijing Acupuncture and Moxibustion Institute for a week and given free access to standard chow pellet diet and water. The rats were randomly divided into three groups: (1) sham/control group: *n* = 15 (sham-operated rats); (2) CCI group, *n* = 13; (3) U0126 (ERK1/2 inhibitor) group, *n* = 15; U0126 (2.26 *μ*L, 10 *μ*g) was injected to the rats. With the automatic injector, the liquid was intracerebroventricularly (i.c.v.) administered within 2 min. All surgical interventions and postoperative animal care were performed in accordance with the Guidelines for Declaration of the National Institutes of Health* Guide for the Care and Use of Laboratory Animals* (publication number 80-23, revised 1996).

### 2.2. Tracheal Intubation and Chronic Constrictive Injury

The rats were anesthetized by a mixture of a solution of urethane (28 mg/100 g) plus chloralose (3.3 mg/100 g). Rectal body temperature was monitored throughout the experiment, and a heating pad was used to maintain the temperature of the animals at 37.0°C ± 0.5°C.

The rats were fixed on the back and the hair on the neck was shaved. The trachea was exposed by blunt dissection through neck muscles after cutting off the neck skin above the manubrium. A T-shaped incision was sheared at the bottom of the thyroid isthmus, and then tracheal intubation was performed using special intubation designed by the laboratory of the Institute of Acupuncture and Moxibustion. In the prone position, the left sciatic nerve was isolated (the chronic constrictive injured nerve in the CCI group) and covered with liquid paraffin.

As described by Bennett and Xie [2], CCI was used as the neuropathic pain model. The left sciatic nerve was exposed and tied around four times by gut suture. The sciatic nerve in the sham group was exposed but not tied. Local application of antibiotics (sodium penicillin, 9000–10000 U/rat) was used to avoid postoperative infection. All models were established by the same experimenter to avoid experimental variability. The electrophysiological experiments were carried out 12 days after the operation.

### 2.3. Recording Neuronal Discharge

The rats were anesthetized by a mixture of urethane (28 mg/100 g) and chloralose (3.3 mg/100 g). The head of the rat was fixed on the stereotaxic apparatus (SR-6R, Nihon Kohden, Tokyo, Japan). With the aid of the stereotaxic atlas of the rat brain, two small skull windows were opened and covered with warm liquid paraffin at the positions for inserting recording electrodes and microsyringe (KDS-310-PLUS, KD Scientific, Holliston, MA, USA). A glass microelectrode (5 MΩ, filled with 3 mol/L KCl) was inserted into the right hippocampal CA1 area (AP: 3.2–3.6 mm; ML: 2.5–3.0 mm; DV: 2.5–3.2 mm) by a micromanipulator (SM-21, Nihon Kohden, Tokyo, Japan) as recording electrode [20]. Another micromanipulator was used to insert the microsyringe into the lateral ventricle (AP: 1.0 mm; ML: 1.3–2.0 mm; DV: 3.0 mm) [21] to inject the experimental drug (MEK1/2 inhibitor). The neuronal discharges were monitored by an oscilloscope (VC-10, Nihon Kohden, Tokyo, Japan) at the same time. After recording spontaneous neural discharge for 5 min as control, the sciatic nerve was stimulated by a double stainless electrode (delay 0, interval 50 msec, duration 0.3 msec, and current intensity 5 mA) for 10 s as noxious stimulation. If there was no change in the neural discharge after noxious stimulation, the neural discharges were not recorded anymore. When the discharge frequency returned to control level (about 10 min after giving the noxious stimulation), the sciatic nerve was given another noxious stimulation for 10 s. At the end of noxious stimulation, bilateral “Zusanli” (ST36) and “Yanglingquan” (GB34) acupoints were punctured with stainless-steel acupuncture needles (gauge 28, 0.20 mm in diameter) to a depth of about 4 mm, respectively, and stimulated electrically using a HANS EA Stimulator for 1 min, and U0126 was administered intracerebroventricularly for 2 min at the same time. “Zusanli” (ST36) and “Yanglingquan” (GB34) are the main points for treating sciatica and other types of leg pain according to the theory of traditional Chinese medicine. Our previous results also suggested that EA at “Zusanli” (ST36) and “Yanglingquan” (GB34) has cumulative analgesic effects for neuropathic pain [14–17]. The complete recording duration was about 30 min.

### 2.4. Definition of Neurons

The response of the neurons to noxious stimuli can have three forms: excitement, inhibition, and no response. Neurons that respond to nociceptive stimulation are defined as pain-related neurons, neurons excited after noxious stimuli are called pain-excited neurons (PENs) [22], and the inhibited neurons are defined as pain-inhibited neurons (PINs) [23]. The discharge frequencies before the noxious stimulus served as control (corresponding to 100%) to observe the changes of discharge frequencies of pain-related neurons. We analyzed the electrical activities of pain-related neurons whose discharge frequencies increased or decreased by more than 20% after noxious stimuli.

### 2.5. Statistical Analysis

The experimental data were scanned on a computer with Spike II (CED Instruments, Cambridge, United Kingdom) after management and analyzed with Spike II software. All data were expressed as mean ± SEM (standard error of the mean). Statistical differences were evaluated by one-way ANOVA. Significance was determined at the level of *P* < 0.05.

## 3. Results

29 pain-related neurons were recorded in the control group (*n* = 15). The electric activities of 17 PENs (58.6%) were increased while those of 12 PINs (41.4%) were decreased after noxious stimuli. In the CCI group (*n* = 13), we recorded 18 pain-related neurons, 14 of which were PENs (78%) and 4 were PINs (22%), so the number of recorded PINs in the control group was larger than in the CCI group. The U0126 group (*n* = 15) showed 15 pain-related neurons, among which there were 9 PENs (60%) and 6 PINs (40%).

### 3.1. EA Regulated the Effects of Acute Noxious Stimulation on the Firing Rates of Pain-Related Neurons in the Hippocampus of Sham Rats

**Figure 1:**
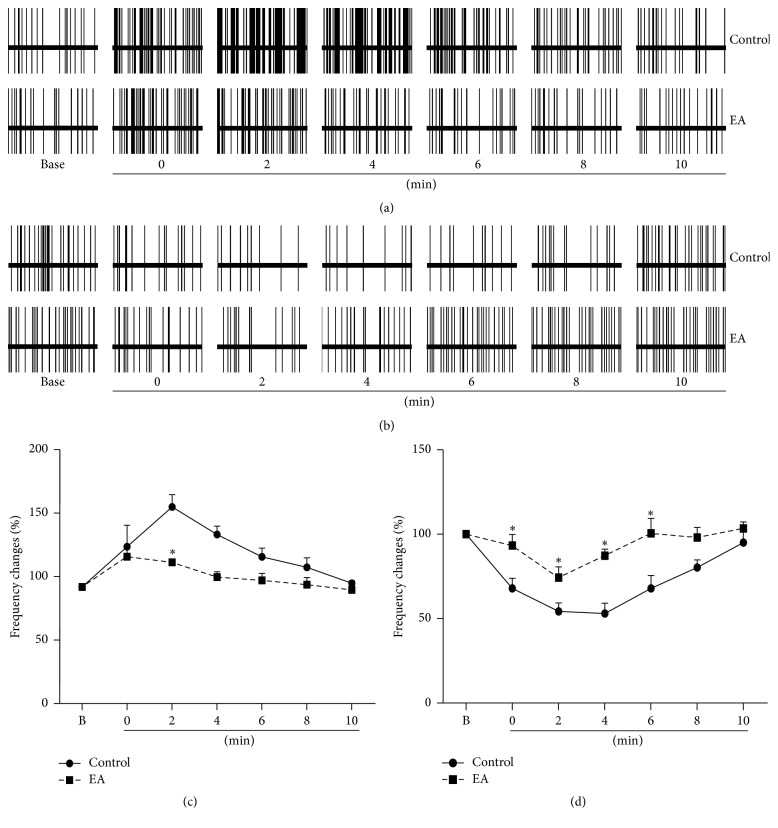
EA reduced the excitatory and inhibitory effects of brief sciatic nerve stimulation on the firing rates of PENs and PINs, respectively, in sham rats. (a) Example of EA reducing the effects of acute noxious stimulation on discharges of hippocampal PENs in the sham group. (b) Example of EA reducing the effects of acute noxious stimulation on discharges of hippocampal PINs in the sham group. (c) EA reduced the effects of acute noxious stimulation on the firing rates of hippocampal PENs (*n* = 17) at different times after the noxious stimuli in sham rats. (d) EA reduced the effects of acute noxious stimulation on the firing rates of hippocampal PINs (*n* = 12) at different times after the noxious stimuli in sham rats. The 10-second stimulation of the sciatic nerve occurred immediately before 0 min, not at 0 min. B on the *x*-axis stands for the background firing rate before the noxious stimulus. *∗*: compared with the control group, *P* < 0.05.

In sham rats, brief sciatic nerve stimulation significantly increased the electrical activities of 17 hippocampal PENs in the control group (134.53 ± 18.50%) and EA group (126.1 ± 8.97%), and there was no difference between the two groups (*P* > 0.05, Figure 1(c)). EA reduced the excitatory effects of brief sciatic nerve stimulation on the firing rates of 17 PENs. At 2 min after the noxious stimuli, the discharge frequency changes of hippocampal PENs in the EA group (121.18 ± 7.45%) were markedly lower than those in the control group (168.68 ± 10.64%, *P* < 0.05). At 4 min after the noxious stimuli, although the firing rates of hippocampal PENs in the control group (145.08 ± 7.22%) were still increased, the firing rates of hippocampal PENs in the EA group (108.65 ± 4.48%) had almost returned to normal level. No significant difference was found between the control group (116.86 ± 8.21%, 103.22 ± 2.13%) and the EA group (102.14 ± 6.03%, 97.65 ± 4.12%) at 8 and 10 min after the noxious stimuli. The electric activities of PENs in the control group almost returned to normal level at 8 min after the noxious stimuli. It is suggested that EA played an inhibiting role in regulating excitatory effects of the acute noxious stimulus on the electrical activity of PENs in sham rats.

The electric activities of 12 hippocampal PINs were decreased by the brief sciatic nerve stimulation in sham rats (Figure 1(d)), and the frequency changes in the control group were larger than those in the EA group. At 0 minutes (immediately) after brief sciatic nerve stimulation, the firing rates in the control group (67.88% ± 6.00) were lower than those in the EA group (93.35% ± 6.46, *P* < 0.05). The frequencies of PINs in the control group were still decreased at 2 min (54.35% ± 4.97), 4 min (53.01% ± 6.12), and 6 min (67.93% ± 7.64) and returned to normal level at 10 min (94.96% ± 6.11) after the stimulation. The frequencies of PINs in the EA group were still at a low level at 2 min (74.36% ± 6.28) and 4 min (87.37% ± 3.78) and returned to normal level at 6 min (100.59% ± 8.80), earlier than in the control group. In comparison with the control group, the frequency changes of PINs in the EA group were higher (*P* < 0.05) at 2, 4, and 6 min after giving the noxious stimuli. Those findings suggested that EA played an exciting role in regulating inhibitory effects of the acute noxious stimulus on the electrical activity of PINs in sham rats.

### 3.2. EA Regulated the Effects of Acute Noxious Stimulation on the Firing Rates of Pain-Related Neurons in the Hippocampus of CCI Rats

**Figure 2:**
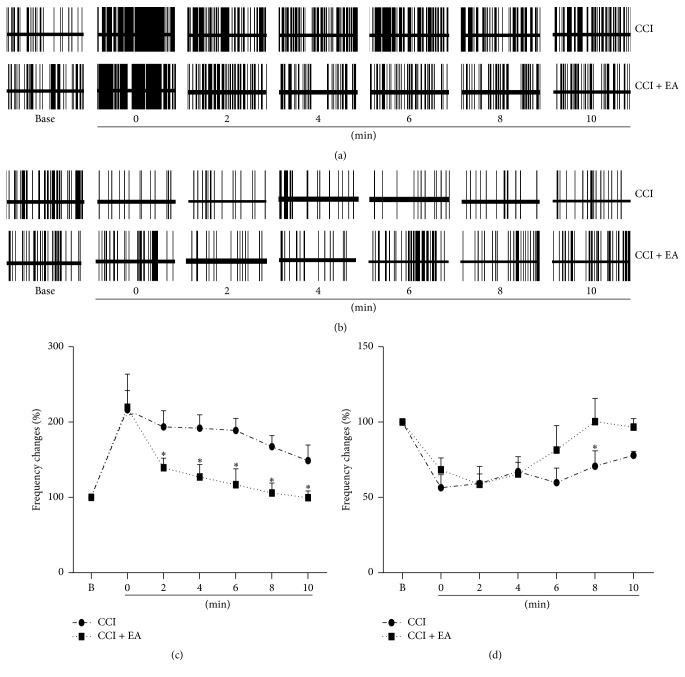
EA reduced the excitatory effects of brief sciatic nerve stimulation on the firing rate of PENs and had a similar reducing effect on PINs in CCI rats. (a) Example of EA reducing the effects of acute noxious stimulation on discharges of hippocampal PENs in CCI rats. (b) Example of EA regulating the effects of acute noxious stimulation on discharges of hippocampal PINs in CCI rats. (c) EA reduced the effects of acute noxious stimulation on the firing rates of hippocampal PENs (*n* = 14) at different times after the noxious stimuli in CCI rats. (d) EA reduced the effects of acute noxious stimulation on the firing rates of hippocampal PINs (*n* = 4) at different times after the noxious stimuli in CCI rats. B on the *X*-axis stands for the background firing rate before the noxious stimulus. *∗*: compared with the CCI group, *P* < 0.05.

The electric activities of 14 hippocampal PENs reached 216.46 ± 25.40% in the CCI group and 219.57 ± 44.15% in the CCI + EA group after the noxious stimulus. There was no significant difference between the two groups (*P* > 0.05, Figure 2(c)) of CCI rats. Similar to the sham rats, the discharge frequencies of 14 PENs were decreased after EA treatment. The frequency changes of hippocampal PENs in the CCI group (193.54 ± 21.50%) were markedly higher than those in the CCI + EA group (139.40% ± 12.78, *P* < 0.05) at 2 min after the noxious stimuli. The firing rates of PENs in the CCI + EA group (116.99% ± 20.90) returned to normal level and were observably lower than those in the CCI group (188.82% ± 16.16, *P* < 0.05) at 6 min. The frequency changes of PENs in the CCI + EA group were 105.87% ± 13.26 and 99.58% ± 9.06 at 8 min and 10 min after the brief sciatic nerve stimulation, which are lower than those in the CCI group (167.27% ± 14.86, 148.82% ± 20.71, *P* < 0.05). It is suggested that EA treatment also reduced the excitatory effects of brief nerve stimulation on the firing rate of PENs in CCI rats.

The discharge frequencies of 4 hippocampal PINs were decreased significantly in both the CCI (56.44% ± 8.68) and the CCI + EA (68.27% ± 7.96) group after brief sciatic nerve stimulation (Figure 2(d)). There was no significant difference between the two groups at 0, 2, and 4 min. At 6 min after giving the noxious stimuli, the frequencies of PINs in the CCI group (59.82% ± 9.57) were still inhibited, and the frequency of PINs in the CCI + EA group (81.40% ± 16.29) had almost returned to normal level. At 8 and 10 min after giving the noxious stimuli, the frequency changes of hippocampal PINs reached 70.65 ± 10.31% and 77.94 ± 2.67%, respectively, in the CCI group; however, the frequencies in the CCI + EA group were still at the normal level (100.30% ± 15.36, 96.69% ± 5.66). The discharge frequency changes of the CCI and the CCI + EA group showed no significant differences at 10 min. Although the number of recorded PINs was small, it is also suggested that EA treatment reduced the effect of brief sciatic nerve stimulation on the frequency of PINs in CCI rats.

### 3.3. Contrasting the Effect of Acute Noxious Stimulation on Frequency Changes of Pain-Related Neurons between Sham and CCI Rats after Noxious Stimuli and EA Treatment

**Figure 3:**
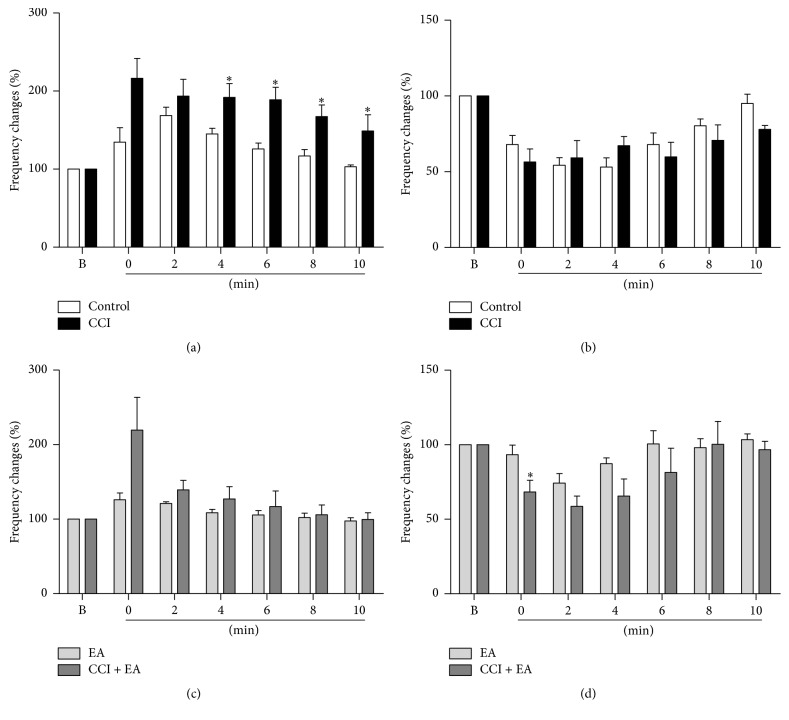
The differences in effects of acute noxious stimulation on the firing rate between sham and CCI rats. The brief sciatic nerve stimulation had a greater excitatory effect on the firing rate of PENs in CCI compared to sham rats (a) and a similar effect on PINs in both CCI and sham rats (b). Also, EA blocked the excitatory effect of the acute noxious stimulus on the firing rate of PENs in both sham and CCI rats (c) and produced slightly less blocking of the inhibitory effect of noxious stimulation on PINs in CCI rats compared to sham rats (d). *∗*: compared with the control group, *P* < 0.05.

The discharges of 17 PENs and 12 PINs were recorded in the hippocampus of 15 sham rats, and those of 14 PENs and 4 PINs were recorded in the CCI rats. At 2 min, there was no difference between sham and CCI rats in the frequency changes (*P* > 0.05, Figure 3(a)); however, brief sciatic nerve stimulation induced bigger frequency changes of PENs in the CCI rats than in the sham rats (*P* < 0.05) from 4 min to 8 min. The discharge frequencies of PENs in the CCI rats were still increased at 10 min, while the discharge frequencies of PENs in the sham rats had almost returned to normal level at 8 min after the acute noxious stimuli.

The discharge frequency changes of PINs in the CCI group were slightly higher than in the control group at 2 and 10 min after the noxious stimuli (*P* > 0.05, Figure 3(b)). This might be associated with the small number of PINs recorded in the CCI group.

Compared with the EA group, the discharge frequency changes of PENs in the CCI + EA group were slightly increased (*P* > 0.05, Figure 3(c)), and those of PINs in the CCI + EA group were mildly decreased (*P* > 0.05, Figure 3(d)) from 2 to 10 min after the noxious stimuli. EA suppressed the excitatory and inhibitory effects of the acute noxious stimulus on the electric activities of PENs and PINs in both sham and CCI rats.

### 3.4. The Effect of the MEK U0126 Inhibitor on EA Treatment in the Hippocampus of Sham Rats

**Figure 4:**
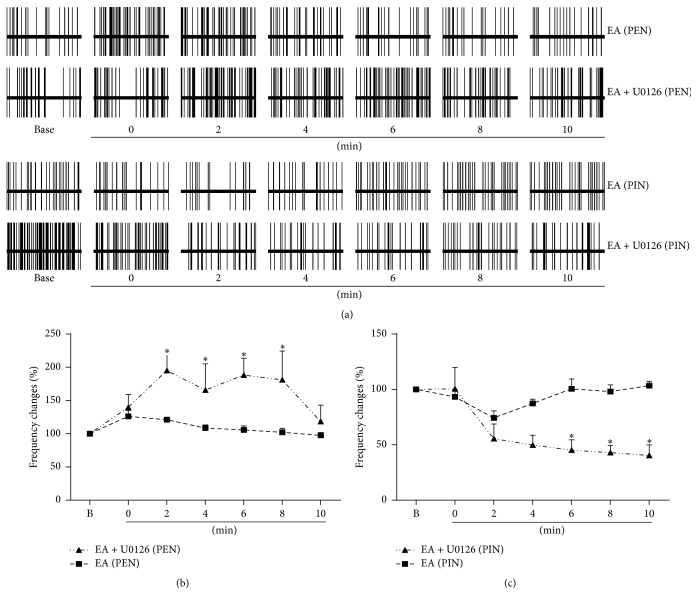
EA was ineffective in the presence of U0126. (a) Example of the effect of U0126 + EA and EA only on the impact of brief sciatic nerve stimulation on the firing rates of hippocampal PENs (*n* = 9) and PINs (*n* = 6). (b) Combination of U0126 and EA increased the discharge frequency changes of PENs caused by acute noxious stimulation to a larger extent than that observed in the EA group. (c) Combination of U0126 and EA decreased the discharge frequency changes of PINs caused by acute noxious stimulation to a larger extent than that observed in the EA group. *∗*: compared with EA group, *P* < 0.05.

Compared with the EA group, the discharge frequency changes of 9 hippocampal PENs in the U0126 group were not significant (*P* > 0.05, Figure 4(b)) at 0 min. Compared with the EA group, the discharge frequency changes of hippocampal PENs in the U0126 group (195.20 ± 22.98%) increased significantly (*P* < 0.05) at 2 min after the noxious stimuli (after injection of U0126). From 4 to 8 min after giving the noxious stimuli, the discharge frequency changes of PENs in the U0126 group (165.80 ± 39.32%, 188.32 ± 25.35%, and 181.14 ± 43.41%) were still higher than those in the EA group (*P* < 0.05). At 10 min after giving the noxious stimuli, no significant differences were found between the U0126 group (118.34% ± 20.80) and the EA group. There was no significant difference between the U0126 group and the EA group in the discharge frequency changes of 6 hippocampal PINs (*P* > 0.05) at 0 min. The discharge frequency changes of hippocampal PINs in the U0126 group (55.6% ± 13.27) were bigger compared to those in the EA group at 2 min after the noxious stimuli (and after injection of U0126). During a period of 6 to 10 min, the discharge frequency changes of PINs in the U0126 group exhibited obvious differences when compared with the same period in the EA group (*P* < 0.05). The electrical activities of PINs gradually returned to baseline levels at 6 min after the noxious stimuli in the EA group, while those in the U0126 group did not. Combination of U0126 + EA in sham rats produced effects markedly greater than those observed when brief sciatic nerve stimulation was performed alone (without drugs), so it may be assumed that U0126 increased the effect of noxious stimulation through a pathway other than that of the EA pathway, thus masking the effect of EA. It is suggested that the MEK1/2 inhibitor U0126 blocked the EA effect on acute noxious stimulation.

## 4. Discussion

Pain is an unpleasant sensory and emotional experience associated with actual or potential tissue damage, or described in terms of such damage. There are two kinds of pain: acute and chronic. Acute pain is very common in clinical practice, including pain in the perioperative setting [24], pain in patients with severe or concurrent medical illnesses (such as arthritis [25]), pain related to cancer or cancer treatment, and labor pain. Acute pain including neuropathic pain [26] affects bodily health and the quality of life. Tens of thousands of people are affected by acute pain, and the treatment costs amount to billions of dollars every year. To enhance the quality of life and maintain the patient's function ability, clinical medicine is used to treat acute pain, but these medications have substantial side effects [27]. Acupuncture analgesia has its advantages, such as an obvious analgesic effect and little side effects, but its mechanism of action is not clarified at the moment. As a part of the limbic system, the hippocampus is associated with memory, but also with pain and acupuncture analgesia. The dorsal hippocampal dopamine receptors exert an analgesic effect during the orofacial pain test [28]; microinjections of nonsteroidal anti-inflammatory drugs [29] or 2-AG [30] into the hippocampus induce antinociception. A recent study indicates that persistent peripheral nociception induced by subcutaneous injection of bee venom upregulated mTOR target p70 S6 kinase signaling and facilitated long-term potentiation which could be reversed by mTOR inhibitor in the hippocampus [31].

In our experiment, we observed that the discharge frequencies of hippocampal CA1 pain-related neurons (PENs or PINs) were changed after brief electrical impulses applied to the sciatic nerve. The electric activities of PENs and PINs returned to normal level at 8 and 10 min after the noxious stimuli, respectively. Xiao et al. [7], Yang et al. [8], Li et al. [9], and Jiao et al. [32] focused on electric activities of pain-related neurons in the hippocampus after noxious stimuli for many years. In the hippocampal CA1 and CA3 area and dentate gyrus, cholinergic neurons and muscarinic receptors have effects on the electric activities of PENs and PINs, so that they are involved in pain modulation [7–9, 32]. Glutamate and its receptors, noradrenaline (NE), phentolamine, and alpha-adrenoceptors also have effects on pain modulation by regulating electric activities of PENs and PINs in the hippocampal CA3 region [33, 34]. These studies reported the effects of pain-induced discharges of hippocampal neurons, but few research papers pay attention to electric activities of hippocampal neurons in a chronic pain state. Hains et al. reported that after spinal cord contusion injury the changes of the spontaneous discharge and afterdischarge of extracellular neurons in the thalamus are related to an upregulated sodium channel expression [35]. In CCI rats, the acute noxious stimulation evoked greater changes of electric activities of PENs and PINs, and the recorded number of hippocampal PENs was obviously bigger and that of PINs smaller compared with sham rats. It took more time for the pain-related neurons to return to normal level in the CCI rats than in sham rats. This might be because the effect of brief sciatic nerve stimulation on firing rates of PENs and PINs was enhanced under the advanced CCI situation. The CCI rats may have experienced more pain than the sham rats after the short noxious stimulation. The CCI rats were in a state of hyperalgesia, an increased response to noxious stimuli, which means the same intensity of electrical stimulation causes more pain intensity and finally a prolonged time of recovery to normal.

Shi et al. [36] reported that pain-related neurons were involved in the modulation of EA analgesia, and EA stimulation resulted in an inhibiting effect on the electrical activity of PENs and an activating effect on the electrical activity of PINs. After EA at the acupoint “Hegu,” or dolantin given intravenously, Gao et al. stimulated the head of the caudate nucleus, eliciting an inhibitory effect on PENs and a reduction of inhibition or release on PINs [37]. EA has been shown to suppress PENs and excite PINs, which can be taken as an electrophysiological index for EA analgesia [38, 39]. Yang et al. also reported that EA treatment could play an inhibiting role in mediating the evoked discharge of PENs and an activating role in that of PINs, and cholecystokinin-8's (CCK-8) B receptor antagonist could be antagonistic to the effect of CCK on EA analgesia [40]. Our results (comp.  Figure 1) showed that, in the hippocampal CA1 area, EA inhibited the excitatory effect of brief sciatic nerve stimulation on the electric activities of PENs and activated the inhibitory effect of brief sciatic nerve stimulation on electric activities of PINs in sham rats, which caused the firing rates of PENs and PINs to return to normal level at 6 min after the noxious stimuli, earlier than in the control group. These findings suggest that the analgesic effect of EA is related to the electric activities of pain-related neurons in the CA1 area, which were affected by noxious stimulation. EA reduced the effect of acute noxious stimulation on the electric activities of PENs and PINs. The electric activities of PENs and PINs play an important role in mediating EA analgesia, as reported before.

In CCI rats, EA also had an effect on the firing rate of PENs and PINs similar to that evoked by acute noxious stimuli, but the electric activities returned to normal level at 6 or 8 min after administration of the noxious stimulus. It is suggested that the effect of EA treatment is closely related to the severity of pain.

Intrathecal administration of the inhibitor of the MAPK family members MEK1/2, such as U0126, PD198306, and PD98059, had analgesic effects and significantly potentiated the effectiveness of opioids in neuropathy in the spinal cord [41–43]. U0126 downregulated the increased late responses and afterdischarge induced by melittin (a pain-related peptidergic component) in wide-dynamic-range neurons of the spinal cord [44]. Accumulating evidence showed that ERK expression increased at the peripheral nerve and spinal cord horn [45–47]. Phospho-ERK (pERK) in the spinal cord is activated immediately in neurons (<6 h), then in microglia on days 1 and 3 and both in astrocytes and in microglia on day 10, and finally in satellite cells on days 10 and 21 after spinal nerve ligation, and this activation contributes to mechanical allodynia [46]. PD 98059 may modulate the nociceptive factors and antinociceptive factors that are released by glial cells, which have a close relationship with reduced symptoms of neuropathy [43, 46]. These reports suggested that MEK1/2 inhibitors have an analgesic effect on neuropathic models in the primary injury and that neuropathic pain is associated with the activity of the MAPK/ERK signal pathway within the spinal cord. Few studies focused on the relationship between the expression of ERK and the hippocampus in chronic neuropathic pain, and an agreement has not been reached [48, 49]. There were no significant changes in hippocampal pERK after formalin injection [50]. Electroacupuncture had inhibiting effects on the ERK signal pathway on the spinal dorsal horn. The phosphorylation of the ERK signal transduction pathway was enhanced by EA in depression rat tissue [51]; however, there was no report on the study of EA analgesia on the hippocampus ERK signal pathway. Our previous results showed that in the hippocampus the ERK signal pathway was inhibited in chronic neuropathic rats, and after acupuncture treatment the ERK signaling pathway was activated [16]. U0126 is a specific inhibitor of MEK, both MEK1 and MEK2. Our experimental results showed that, compared with the EA group, brief sciatic nerve stimulation produced a greater excitatory and inhibitory effect on the firing rate of PENs and PINs in U0126 + EA group, suggesting that U0126 suppressed the effect of EA on the analgesic pathway. It is suggested that involvement of the activation of ERK signaling pathway in the hippocampal CA1 region in EA treatment induced pain relief.

## 5. Conclusions

In CCI rats, acute noxious stimulation required a longer time for the firing rate of pain-related neurons to return to normal level; EA treatment could suppress the effect of the noxious stimulus on PENs and PINs in both sham and CCI rats, which suggests a close relationship with the EA analgesic effect; and the ERK signal pathway is probably involved in pain and EA analgesia.
